# Association of Vitamin D Receptor BsmI Gene Polymorphism with Risk of Tuberculosis: A Meta-Analysis of 15 Studies

**DOI:** 10.1371/journal.pone.0066944

**Published:** 2013-06-25

**Authors:** Yu-jiao Wu, Xin Yang, Xiao-xiao Wang, Man-Tang Qiu, Yi-zhong You, Zhi-xin Zhang, Shan-mei Zhu, Lin Xu, Feng-lei Tang

**Affiliations:** 1 Department of Pharmacy, Changzhou Third People’s Hospital, Changzhou, China; 2 College of Pharmacy, Soochow University, Suzhou, China; 3 The Fourth Clinical College of Nanjing Medical University, Nanjing, China; 4 Department of Thoracic Surgery, Nanjing Medical University Affiliated Cancer Hospital Cancer Institute of Jiangsu Province, Nanjing, China; 5 Department of Bio-statistics, Georgia Health Science University, Augusta, Georgia, United States of America; 6 Department of Pharmacy, Changzhou First People’s Hospital, Changzhou, China; 7 Department of Pulmonary Tuberculosis, Changzhou Third People’s Hospital, Changzhou, China; Fundacion Huesped, Argentina

## Abstract

**Background:**

Genetic variations in vitamin D receptor (VDR) may contribute to tuberculosis (TB) risk. Many studies have investigated the association between VDR BsmI gene polymorphism and TB risk, but yielded inconclusive results.

**Methodology/Principal Findings:**

We performed a comprehensive meta-analysis of 15 publications with a total of 2309 cases and 3568 controls. We assessed the strength of the association between VDR BsmI gene polymorphism and TB risk and performed sub-group analyses by ethnicity, sample size and Hardy–Weinberg equilibrium (HWE). We found a statistically significant correlation between VDR BsmI gene polymorphism and decreased TB risk in four comparison models: allele model (b vs. B: OR = 0.78, 95% CI = 0.67, 0.89; P_heterogeneity_ = 0.004), homozygote model (bb vs. BB: OR = 0.61, 95% CI = 0.43, 0.87; P_heterogeneity_ = 0.001), recessive model (bb vs. Bb+BB: OR = 0.70, 95% CI = 0.56, 0.88; P_heterogeneity_ = 0.005) and dominant model (bb+Bb vs. BB: OR = 0.77, 95% CI = 0.61, 0.97; P_heterogeneity_ = 0.010), especially in studies based on Asian population. Sub-group analyses also revealed that there was a statistically decreased TB risk in “small” studies (<500 participants) and studies with P_HWE_>0.5. Meta-regression and stratification analysis both showed that the ethnicity and sample size contributed to heterogeneity.

**Conclusions:**

This meta-analysis suggests that VDR BsmI gene polymorphism is associated with a significant decreased TB risk, especially in Asian population.

## Introduction

According to the latest information of the tuberculosis (TB) epidemic provided by the World Health Organization (WHO) Global Tuberculosis Report 2012, the TB mortality and incidence rates have been decreasing for several years. However, the global burden of TB remains enormous, especially in South-East Asia and South Africa, the number of new cases in 2011 is 8,700,000 including 1,131,000 HIV-infected patients; and 1,400,000 people died from TB, including 1,000,000 HIV-negative patients and 430,000 HIV-positive patients [1]. Although it is estimated that about one third of the world’s population is infected with bacillus which may cause TB, a relatively small proportion of people (10%) infected with bacillus will progress to active TB disease [2]. It is suggested that the susceptibility to TB is influenced by many factors, such as HIV infection, environmental and host genetic factors [3]–[6]. Recently, there are various studies reporting that host genetic factors may play an important role in susceptibility to TB. Multiple candidate genes have been investigated to determine the relationship between single nucleotide polymorphisms (SNPs) and TB risk, including the natural resistance–associated macrophage protein 1 (NRAMP1) gene [7], interleukin (IL) genes [8], [9], vitamin D receptor (VDR) genes [10], and tumor necrosis factor (TNF) genes [11].

The interaction of 1,25-dihydroxyvitamin D3 with the vitamin D receptor (VDR) is able to activate monocytes, stimulate cell-mediated immunity and suppress lymphocyte proliferation [12], therefore the susceptibility to TB may be increased by deficiency of 1,25-dihydroxyvitamin D3 [13]. Vitamin D also play a key role through VDR gene mutating that may affect the immunity activity and the subsequent mediated effect of VDR, it has been considered as a risk factor in TB development process. One of the most frequently studied VDR polymorphisms is BsmΙ which is in intron 8 binding to the 3′UTR, and it is genotyped as BB, Bb, or bb. A lot of investigations are performed to explore the association of VDR BsmI gene polymorphism with TB risk, but the findings are conflicting. For example, in 2004, Bornman found that there was no statistically significant association between TB and VDR BsmI gene polymorphism by a case-control analysis [14]. However, Ates reported that VDR BsmI gene bb genotype could significantly decrease TB risk in 2011 [15]. In 2010, a large system review suggested the BsmI polymorphism was related to TB risk only in Asians [16], but it failed to identify a significant result of BsmI polymorphism in overall populations.

To confirm the association between vitamin D receptor BsmI gene polymorphism and TB risk, we performed this meta-analysis by calculating the estimate of overall TB risk and evaluated influence of ethnicity, sample size and Hardy–Weinberg equilibrium (HWE).

## Methods

### Literature Search Strategy

According to the Preferred Reporting Items for Systematic Reviews and Meta-Analyses (PRISMA), a systematic literature searching was performed on PubMed, EMBASE and Chinese National Knowledge Infrastructure (CNKI) up to March 19, 2013. In order to identify as many relative articles as possible, the search strategy was based on combinations of “Vitamin D receptor” or “VDR”; “BsmI”, “rs1544410”; “polymorphism”, “variant”, “locus” or “SNP”; “tuberculosis”. Furthermore, we performed manual search of references of relative articles and reviews. To minimize potential publication bias, no restrictions were placed on language, sample size and time period.

### Inclusion and Exclusion Criteria

Studies were selected according to the following inclusion criteria: (a) investigation on the association of VDR BsmI gene polymorphism with TB risk; (b) case-control studies; (c) genotype distribution information in cases and controls; (d) genotype frequencies found in the control group deviate from Hardy-Weinberg equilibrium (HWE). The major reasons for exclusion of studies were (a) overlapping data; (b) case-only studies, reviews, letters and editorials; (c) studies without detailed BsmI genotype frequencies.

### Data Extraction

Data of each eligible study was extracted by two reviewers (Wu and Yang) independently with a standard data-collection method. The following data was extracted: first author, year, country, ethnicity, source of controls, TB diagnosis standards, number of cases and controls, genotype frequency in cases and controls. Different ethnicities were categorized as Asian and African. According to the source of controls, all eligible studies were defined as hospital-based (HB) and population-based (PB). The Hardy–Weinberg equilibrium (HWE) was examined by Chi-square test (p<0.05 was considered as significant disequilibrium) based on BsmI genotyping distribution in controls [17].

### Statistical Analysis

The strength of the association between VDR BsmI gene polymorphism and TB risk was evaluated by calculating pooled odds ratio (OR) with 95% confidence intervals (95% CI). The pooled ORs were calculated for five comparison models: allele model (b vs. B), homozygote model (bb vs. BB), heterozygote model (Bb vs. BB), dominant model (bb+Bb vs. BB) and recessive model (bb vs. Bb+BB). The statistical heterogeneity between studies was checked using Chi-square based Q test and considered significant at P<0.1 [18]. When there was no significant heterogeneity, the fixed effects model (Mantel-Haenszel method) was used [19]; otherwise, the random-effects model (the Der Simonian and Laird method) was utilized [20]. Sensitivity analyses were performed to identify individual study’s effect on pooled results and test the reliability of results [17]. Stratification and logistic meta-regression analyses were performed to explore the source of heterogeneity among variables, such as ethnicity and sample size (studies with more than 1000 participants were defined as “large”, and studies with less 1000 participants were defined as “small”). Publication bias was both examined with Egger’s test and Begg’s funnel plot, and the statistical significance was defined as P<0.05 [21]. All P values are two-sided. Data were analyzed using STATA software (version 12.1; Stata Corp, College Station, Texas USA).

## Results

### Characteristics of All Included Studies

**Figure 1:**
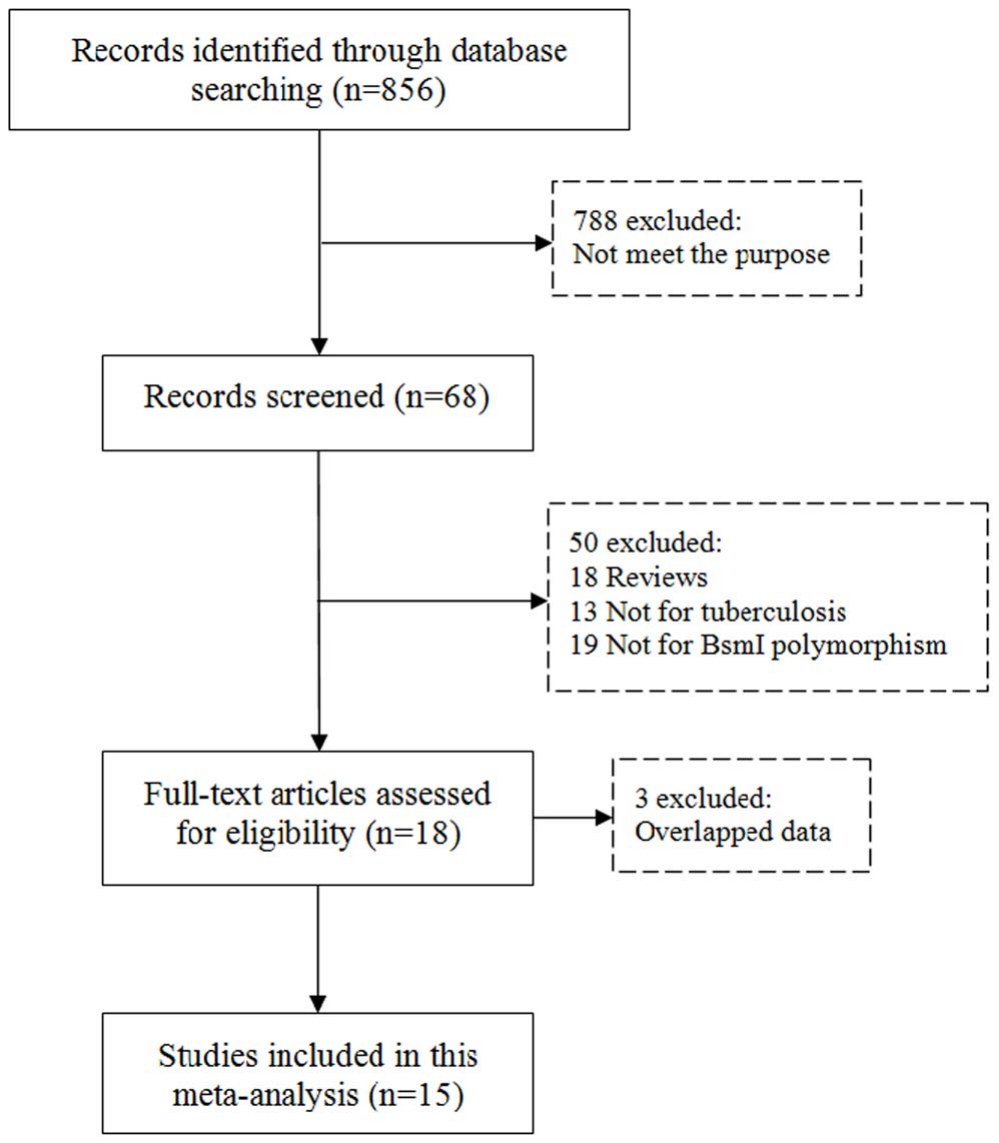
PRISMA Flow Chart.

Study selection process was shown in Figure 1. A total of 15 case-control studies [14]–[15], [22]–[34], including 2309 cases and 3568 controls, were identified according to inclusion and exclusion criteria. Among the 15 eligible studies, 4 of them were studies of Africans [14], [22], [24], [25], and 11 studies were of Asians [15], [23], [26]–[34]. Furthermore, the eligible studies also contained 4 “large” studies [14], [22], [25], [32]and 11 “small” studies [15], [23], [24], [26]–[31], [33], [34]. When we performed HWE test on controls of each study, it showed that there were 8 studies with P_HWE_>0.05 [14], [15], [22], [23], [25], [27], [28], [34], and 7 studies with P_HWE_<0.05 [24], [26], [29]–[33]. The detailed characteristics of the eligible studies included in this meta-analysis are shown in Table 1.

**Table 1:** Characteristics of Eligible Studies.

Firstauthor	Year	Country	Ethnicity	Control	Diagnosis standards	HIVInfection	HWE	Cases			Controls		
								BB	Bb	bb	BB	Bb	bb
**Fitness**	2004	Malawi	African	PB	Bacteriology	Positive	Yes^a^	212	123	10	314	192	39
**Selvaraj**	2004	India	Asian	PB	Clinical symptoms, bacteriology,X-ray	Negative	Yes	16	24	6	18	27	19
**Bornman**	2004	West Africa	African	PB	Clinical symptoms, bacteriology,X-ray	Mixed	Yes	20	108	215	39	208	387
**Lombard**	2006	South Africa	African	HB	Clinical symptoms, bacteriology	Negative	No^b^	6	38	60	9	32	76
**Olesen**	2007	West Africa	African	PB	Clinical symptoms, bacteriology	Mixed	Yes	146	141	33	152	152	38
**Selvaraj**	2008	India	Asian	PB	Clinical symptoms, bacteriology,X-ray	Negative	No	23	16	12	16	17	27
**Alagarasu**	2009	India	Asian	PB	Clinical symptoms, bacteriology,X-ray	Positive	Yes	40	47	20	39	62	45
**Selvaraj**	2009	India	Asian	PB	Clinical symptoms, bacteriology,X-ray	Negative	Yes	27	22	16	16	23	21
**Vidyarani**	2009	India	Asian	PB	Clinical symptoms, bacteriology,X-ray	NA	No	16	14	10	15	13	21
**Merza**	2009	Iran	Asian	PB	Clinical symptoms, bacteriology,X-ray	NA	No	7	67	43	13	21	26
**Marashian**	2010	Iran	Asian	PB	Bacteriology,X-ray	NA	No	23	86	55	0	29	21
**Sharma**	2011	India	Asian	PB	Clinical symptoms, bacteriology,X-ray	NA	No	73	102	53	274	568	211
**Singh**	2011	India	Asian	PB	Clinical symptoms, bacteriology,X-ray, PPD	Negative	No	32	52	17	57	134	34
**Ates**	2011	Istanbul	Asian	PB	Clinical symptoms, bacteriology,X-ray	NA	Yes	28	68	32	5	38	37
**Kang**	2011	Korean	Asian	PB	Bacteriology	NA	Yes	2	13	135	0	8	75

PB: population-based; HB: hospital-based; a: studies with P_HWE_>0.05; b: studies with P_HWE_<0.05.

### Meta-analysis Results

**Figure 2:**
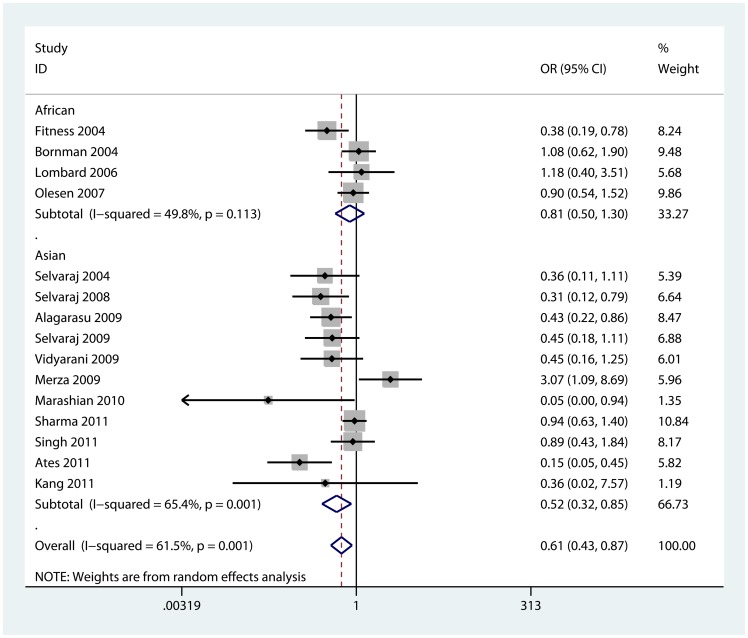
Forest plot of homozygote model for overall comparison (bb vs. BB).

**Figure 3:**
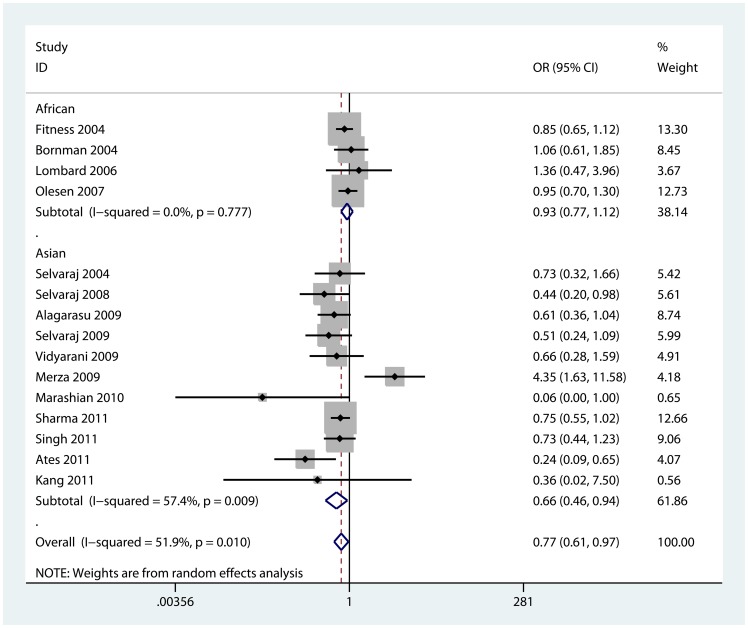
Forest plot of dominant model for overall comparison (bb+Bb vs. BB).

We observed a significant decreased risk of TB susceptibility in allele model (b vs. B: OR = 0.78, 95% CI = 0.67, 0.89; P_heterogeneity_ = 0.004), homozygote model (bb vs. BB: OR = 0.61, 95% CI = 0.43, 0.87; P_heterogeneity_ = 0.001, Figure 2), recessive model (bb vs. Bb+BB: OR = 0.70, 95% CI = 0.56, 0.88; P_heterogeneity_ = 0.005, Figure S1) and dominant model (bb+Bb vs. BB: OR = 0.77, 95% CI = 0.61, 0.97; P_heterogeneity_ = 0.01, Figure 3). However, no significant association except a decreased trend was found in heterozygote model (Bb vs. BB: OR = 0.86, 95% CI = 0.67, 1.09; P_heterogeneity_ = 0.016). The association strength between VDR BsmI gene polymorphism and TB risk is shown in Table 2.

**Table 2:** Meta-analysis Results.

		b vs. B		bb vs. BB		Bb vs. BB		bb+Bb vs. BB		bb vs. Bb+BB	
	N	OR	P_h_	OR	P_h_	OR	P_h_	OR	P_h_	OR	P_h_
**Total**	15	0.78(0.67,0.89)*	0.004	0.61(0.43,0.87)*	0.001	0.86(0.67,1.09)	0.016	0.77(0.61,0.97)*	0.010	0.70(0.56,0.88)*	0.005
**Ethnicities**											
**African**	4	0.92(0.81,1.06)	0.351	0.81(0.50,1.30)	0.113	0.98(0.80–1.20)	0.769	0.93(0.77,1.12)	0.777	0.80(0.54,1.16)	0.055
**Asian**	11	0.70(0.57,0.85)*	0.01	0.52(0.32,0.85)*	0.001	0.77(0.53,1.12)	0.012	0.66(0.46,0.94)*	0.009	0.65(0.49,0.88)*	0.015
**Sample Size**											
**Large** a	4	0.94(0.84,1.05)	0.336	0.82(0.56,1.19)	0.116	0.88(0.73,1.05)	0.353	0.86(0.73,1.02)	0.622	0.92(0.65,1.30)	0.038
**Small** b	11	0.68(0.56,0.82)*	0.068	0.51(0.31,0.86)*	0.005	0.84(0.53,1.33)	0.007	0.68(0.44,1.03)	0.006	0.61(0.49,0.77)*	0.346
**HWE**											
**Yes** c	8	0.75(0.61,0.92)*	0.009	0.50(0.32,0.79)*	0.020	0.89(0.74,1.06)	0.497	0.76(0.60,0.97)*	0.150	0.63(0.44,0.90)*	0.007
**No** d	7	0.80(0.64,1.00)	0.050	0.78(0.45,1.37)	0.013	0.99(0.55,1.78)	0.002	0.85(0.51,1.40)	0.005	0.78(0.57,1.07)	0.080

N: number of studies included; OR: odds ratio; P_h_: p value for heterogeneity;

* OR with statistical significance;

a :studies with more than 1000 participants;

b :studies with less than 1000 participants;

c :studies with P_HWE_>0.05;

d :studies with P_HWE_<0.05.

Furthermore, we performed sub-group analyses to explore the effect of ethnicity, sample size and HWE. As for ethnicities, a decreased TB risk was found in Asian population in four comparison models: allele model (b vs. B: OR = 0.70, 95% CI = 0.57, 0.85; P_heterogeneity_ = 0.010), homozygote model (bb vs. BB: OR = 0.52, 95% CI = 0.32, 0.85; P_heterogeneity_ = 0.001), recessive model (bb vs. Bb+BB: OR = 0.65, 95% CI = 0.49, 0.88; P_heterogeneity_ = 0.015) and dominant model (bb+Bb vs. BB: OR = 0.66, 95% CI = 0.46, 0.94; P_heterogeneity_ = 0.009). In Africans, however, no significant association except only a reduced trend was found in each model.

When stratified by sample size, we found a decreased risk of TB in “small” studies for three comparison models: allele model (b vs. B: OR = 0.68, 95% CI = 0.56, 0.82; P_heterogeneity_ = 0.068), homozygote model (bb vs. BB: OR = 0.51, 95% CI = 0.31, 0.86; P_heterogeneity_ = 0.005), and recessive model (bb vs. Bb+BB: OR = 0.61, 95% CI = 0.49, 0.77; P_heterogeneity_ = 0.346). In a coincidence, three studies [14], [22], [25] based on Africans were“large” studies, so we also found an insignificant result in “large” studies.

Further, in the stratified analyses by HWE, we observed the BsmI polymorphism was correlated with a decreased risk of TB in studies with P_HWE_>0.05 for four comparison models: allele model (b vs. B: OR = 0.75, 95% CI = 0.61, 0.92; P_heterogeneity_ = 0.009), homozygote model (bb vs. BB: OR = 0.50, 95% CI = 0.32, 0.79; P_heterogeneity_ = 0.020), recessive model (bb vs. Bb+BB: OR = 0.63, 95% CI = 0.44, 0.90; P_heterogeneity_ = 0.007) and dominant model (bb+Bb vs. BB: OR = 0.76, 95% CI = 0.60, 0.97; P_heterogeneity_ = 0.150).

### Evaluation of Heterogeneity

Heterogeneity between studies in each model is shown in Table 2. We investigated the source of heterogeneity by publication years, ethnicity, source of controls and sample size with meta-regression. Meta-regression results revealed that ethnicities and sample size (p<0.05), but not publication years (p = 0.913) and source of controls (p = 0.609) were the sources of heterogeneity. When existed significant heterogeneity we adopted random-effects model; otherwise, we adopted fixed-effects models.

### Sensitivity Analyses and Publication Bias

**Figure 4:**
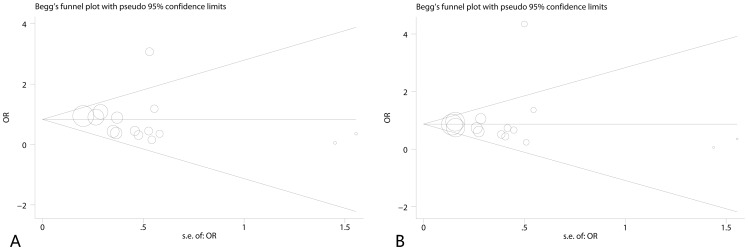
Funnel plot analysis to detect publication bias in 15 eligible studies A: Funnel plot analysis of homozygote model (bb vs. BB). Egger’s test p = 0.617, Begg’s test p = 0.921; B: Funnel plot analysis of dominant model (bb+Bb vs. BB). Egger’s test p = 0.685, Begg’s test p = 1.000; the circles represent the weight of individual study.

Sensitivity analysis was performed to assess the influence of each individual study on the pooled OR by deleting one single study each time. The results showed that no individual study affected the pooled OR significantly, suggesting stability of this meta-analysis (Figure S2). The Begg’s funnel plot and the Egger’s test were used to assess publication bias. The Begg’s funnel plot seemed symmetrical (Figure 4). Furthermore, the statistical results still showed no publication bias by Egger’s test in eligible studies(p>0.05).

## Discussion

Tuberculosis is second only to HIV as the greatest killer due to a single infectious agent worldwide. Gene mutation could affect the function of VDR gene and might be associated with TB risk. In recent years, given the potential roles of VDR playing in the etiology of TB, more studies have been conducted to identify whether the VDR BsmI gene polymorphism was the genetic determiner of TB. However, these studies yielded different or even controversial results. In this meta-analysis, we found that the b allele was associated with a significant decreased risk of TB in four main comparison models. Therefore, it was first reported that VDR BsmI gene polymorphism could predict the susceptibility of TB.

Linkage disequilibrium measure is used to describe the association of alleles of adjacent polymorphisms with each other. BsmI (B>b), ApaI(A>a) and TaqI(T>t) are located near the 3′UTR of VDR gene through the strong linkage disequilibrium, and the extended genotype baT is associated with increased level of VDR [35]. The 3′UTR of VDR gene is known to be involved in regulation of mRNA stability. In 1994, Morrison first reported allelic differences of VDR gene including BsmI polymorphism in the 3′ UTR might alter mRNA levels [36]. Whitfield and colleagues verified that the baT genotype could increase VDR gene expression and mRNA levels compared with BAt genotype in human osteoblast cell line [37]. Verbeek also found that the baT genotype contributed to high levels of VDR gene expression in human peripheral blood lymphocytes, leukemia cell line, prostate cell line [38]. On the other hand, Vitamin D not only regulates the metabolism of calcium and phosphorus, it also acts as an important immune gene regulating hormone with its metabolism function closely related to the macrophage activity [39]. When 1,25-dihydroxyvitamin D3 combined with VDRs that scattered on the surface of lymphocytes and monocytes, it could stimulate the immune response of activating monocyte and turn its prosoma into its effective form, maintain its adhesive capacity, and strengthen its lethal effect to *Mycobacterium tuberculosis*. The exposure to 1,25-dihydroxyvitamin D3 metabolites in vitro was able to increase the ability of monocytes to control proliferation of *Mycobacterium tuberculosis* [40]. Therefore, the BsmI polymorphism of VDR gene could influence the activity of acceptor, and it was considered to be a potential sign of host’s susceptibility to TB.

In this meta-analysis, we demonstrated that VDR BsmI gene polymorphism (B>b) contributed to TB risk in allele model (b vs. B), homozygote model (bb vs. BB), recessive model (bb vs. Bb+BB) and dominant model (bb+Bb vs. BB), which was in inconsistent with several previous independent studies [14], [23]–[25], [32]–[34]. Most studies suggested that a trend of reduced TB risk was observed in bb genotype compared with BB genotype, while an opposite trend was found in a study based on Iranian population which indicated that the bb genotype could increase TB risk on the contrary [30]. It may derive from different experimental designs or methods, which call for further investigation.

As we know, gene polymorphisms are complicated and fluctuating, which mainly attributed to different ethnicities. Moreover, the burden of TB is highest in Asia and Africa geographically. Therefore, we performed a sub-group analysis on ethnicities in this meta-analysis. It showed that the VDR BsmI gene polymorphism had a decreased risk of TB in Asians rather than Africans, especially in the same four comparison models above mentioned (Table 2). However, an insignificant association was found in Africans for all comparison models. To a certain extent, this finding could reflect the existence of racial differences. Previous studies including WHO tuberculosis report suggested that the yellow race was more susceptible to TB than the black and white race [41]. It might be owing to several environmental factors which were able to influence the levels of VDR gene expression on different populations, including dietary habits, intensity and hours of sunlight. Additionally, it was reported that the b allele frequency was higher in Asians than Caucasians and Africans [42]. Thus, the current finding of this meta-analysis might attribute to the racial differences.

During other sub-group analyses, we also found that sample size greatly affected the association between VDR BsmI gene polymorphism and TB risk. As shown in Table 2, there was a significantly decreased TB risk of three genotypes of VDR BsmI gene polymorphism in “small” studies, but insignificant association except only a decreased trend was found in “large” studies. It was worth noting that, there were three papers [14], [22], [25] based on Africans in all four“large” studies.

HWE is essential for a sound case–control study. It is probable that studies without HWE in controls have selection bias or genotyping error, which may cause misleading results. In this meta-analysis, we found the BsmI polymorphism was significantly associated with a decreased risk of TB in studies with P_HWE_>0.05. And five studies with P_HWE_>0.05 were based on Asians [15], [23], [27], [28], [34]. Thus, it is reasonable that racial differences are more likely to explain the results of sub-group analyses than sample size and HWE. More“large” studies in agreement with HWE based on Asians are required.

For heterogeneity, we found ethnicity and sample size were the source of heterogeneity, which was in consistent with sub-group analyses. When studies were stratified by ethnicity and sample size, we conducted heterogeneity test to explore the source of heterogeneity. The obvious heterogeneity existed in Asians and “small” studies. The racial differences might be responsible for the foremost source of heterogeneity. In addition, the “small” studies may be affected by the small-study effect, in which effects reported are larger, and lead to between studies variance. Thirdly, we observed that eligible studies were conducted in seven countries in Asia and Africa respectively. The environmental factors which may influence VDR gene expression are quite different in these countries. For example, the intensity and hours of sunlight in South Africa are stronger and longer than Korean by geography. Fourthly, the different experimental designs, diagnosis standards, HIV status and even socialized medical care also may contribute to the heterogeneity. Thus this kind of heterogeneity is hard to exclude, because recruitment of enough TB cases came from specific regions is difficult.

As for the aforementioned publication bias detected by Begg’s funnel plot and Egger’ test, the funnel plot was roughly symmetrical and no publication bias had been detected. Although Merza’s study was the only study reported increased TB risk in homozygote, heterozygote and dominant model, the effect for the publication bias was small. So we had not ruled out the Merza’s study. The results of this meta-analysis are relatively stable and reliable.

Although a previous meta-analysis conducted by Gao et al suggested the BsmI polymorphism was related to host susceptibility to TB in Asians [16], evidence for overall populations was still insignificant. In our meta-analysis, the number of studies included was almost twice as that reported by Gao et al (15 vs. 9), which can provide enough statistical power to detect modest difference. We first reported that the VDR BsmI gene polymorphism was associated with decreased TB risk in overall populations. Additionally, there are some limitations which need to be addressed. First, although all most control sources were population-based which might be represent of the general population, we could not obtain enough individual information and a more precise adjusted OR for other covariates such as age, sex and environment factors was not allowed. Secondly, the number of studies based on Africans and “large” studies based on Asians were too small.

In conclusion, results from this meta-analysis demonstrate that VDR BsmI gene polymorphism is associated with decreased TB risk, especially in Asian population. To confirm this association, future large scale case-control studies are required to validate these findings.

## Supporting Information

Figure S1 **Forest plot of recessive model for overall comparison (bb vs. Bb+BB).**
(TIF)

Figure S2 **Sensitivity Analyses.** The pooled odds ratios were calculated by omitting each data set at a time.
(TIF)

Checklist S1 **PRISMA checklist.**
(DOC)
