# Supplementary figures and images for: Association of Vitamin D Receptor BsmI Gene Polymorphism with Risk of Tuberculosis: A Meta-Analysis of 15 Studies
Source: PLoS One. 2013 Jun 25;8(6):e66944. doi: 10.1371/journal.pone.0066944 (PMC3692555; doi:10.1371/journal.pone.0066944)

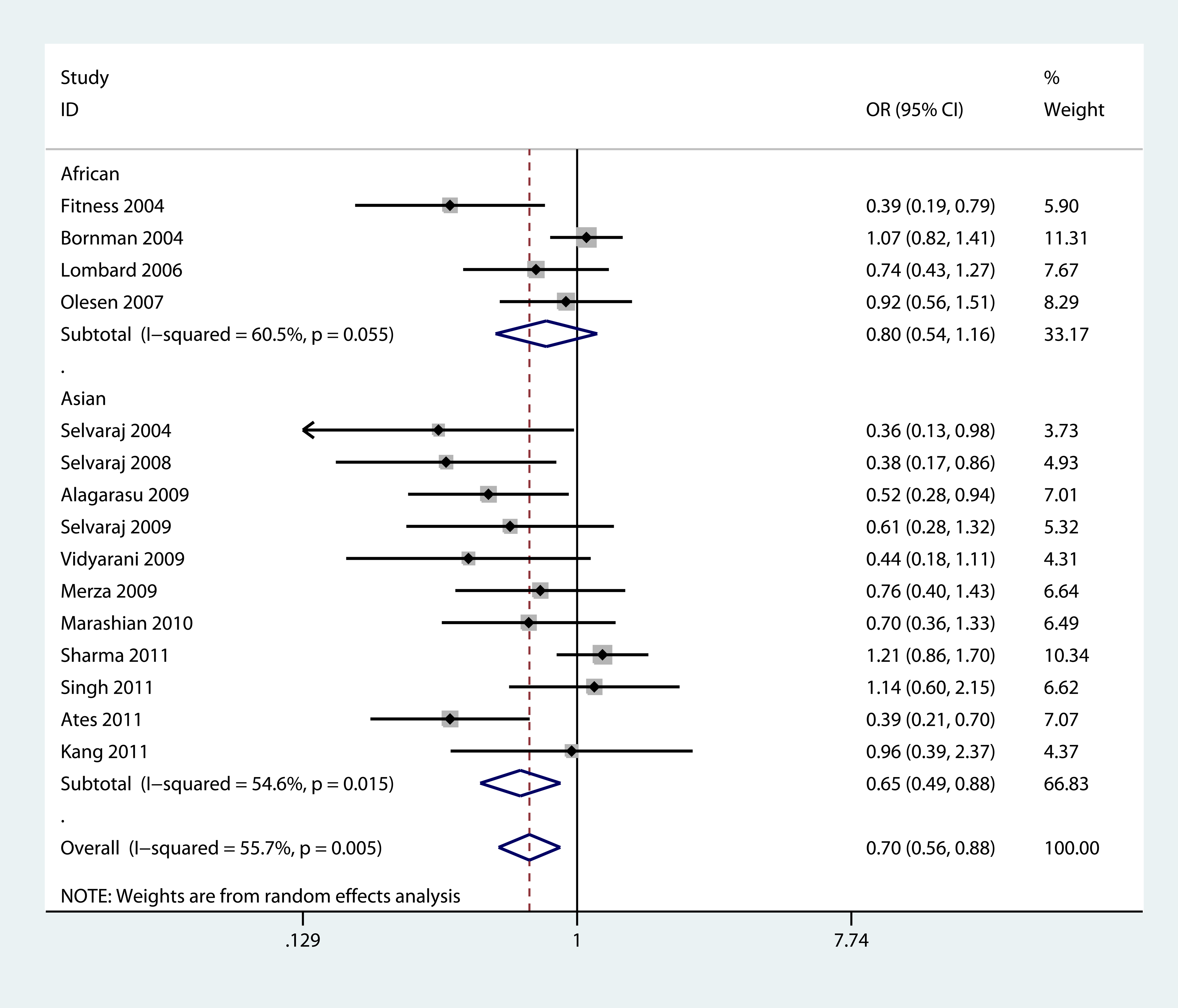

Supplement: Figure S1 — Forest plot of recessive model for overall comparison (bb vs. Bb+BB). (TIF) [file pone.0066944.s001.tif]

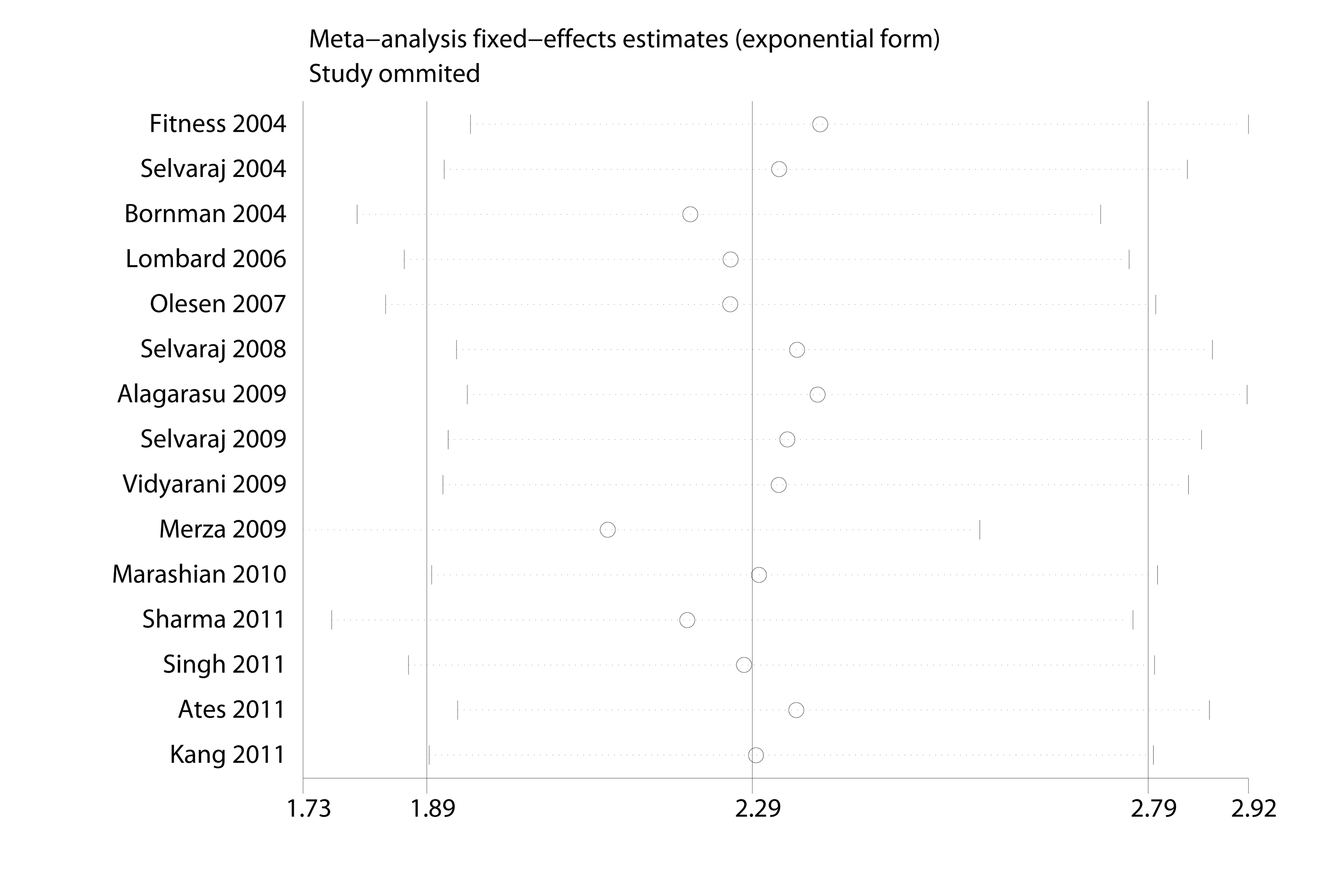

Supplement: Figure S2 — Sensitivity Analyses. The pooled odds ratios were calculated by omitting each data set at a time. (TIF) [file pone.0066944.s002.tif]
